# Syphilis and Its Treatment

**Published:** 1910-09

**Authors:** M. H. Simons

**Affiliations:** Medical Director U. S. N.


### Abstracts and Selections.

### Syphilis and Its Treatment.

It is not the intention of this article to make a study of syphilis, but to discuss it briefly, with the view of arousing more active interest in the management of a disease which causes so much suffering and disfigurement and adds so largely to the annual total of our sick days.

As might be expected with the increase in population and the overcrowding of cities, the venereal diseases, as well as tuberculosis, are increasing. According to reports published in the medical papers, syphilis is increasing in European countries also. There are fewer soft ulcers, in proportion, and more of the hard. On my last cruise, which covered pretty thoroughly both coasts of the United States and South America, the conclusion was forced upon me that constitutional infection followed more frequently than formerly soft sores and abrasions; these would partly heal and then induration of the base and constitutional symptoms would follow.

In 1494 the disease became epidemic in parts of Spain and, following the arrival of a Spanish army in Italy, it became prevalent to a frightful extent there among all classes, and spread to other countries. The Spanish called it the "American disease," claiming that their soldiers contracted it from the aborigenes of the West Indies, and it was called also the "French disease" and laid to the French soldiers in Italy. Other diseases marked by eruptions and ulcerations have long been confused with syphilis, and the diagnosis has been so uncertain that it is impossible to determine when and where the disease arose. It is said that the Mexicans and Peruvians recognized the disease and had separate burial rites for those dying of it; also that the Peruvian name for it was "Huanti." It is also stated that the Chinese described, about 4000 B. C., four different diseases which, taken together, form a proper picture of syphilis and that these diseases were treated by the mercurial earths and decoction of seaweed. It is quite certain that syphilis has been common in China for a long period. The Japanese also recognized it early in their history. As the Mongol peoples were in contact with western peoples in Central Asia (Babylon, Persia, Asia Minor) in very ancient times, it is fair to presume that venereal diseases spread from one to the other. It is also suggested, in line with evolution, that the form of treponema causing syphilis and those forms found in other diseases may have been developing their differences slowly, and as these have become more fixed the results of their activity have become more characteristically certain and more pronounced.

The outbreak of the disease over so great an extent of territory in 1495 caused the medical men of all nations to concentrate their faculties upon its treatment. In 1495 mercury was announced as a cure; from whom or from whence the knowledge was derived is unknown, but it seems likely that it had long been used by some, and general attention was simply called to this usage. Its overuse produced such bad effects that medical men soon became divided into mercurialists and anti-mercurialists, but nothing has been found to take its place, and it has gradually assumed the rank of a specific. Thirty-odd years ago erosions of both soft and bony tissues from mercurial poisoning were not at all uncommon. As calomel was the form of drug then used, it has fallen into unmerited disrepute, and the graduate of the present day hesitates to use the most generally useful of the mercurial preparations. At the clinic of the Presbyterian Hospital in Canton, China, natives would present themselves with cases of the most frightful necrosis of the soft and bony tissues. It was thought by the foreign medical men that this was caused by the prolonged use of cinnabar, or some other natural mercurial earth. One could readily understand, after seeing these cases, why there was formerly such strong opposition to mercury. But the abuse of a remedy is no argument against its legitimate use.

The recognition of mercury as the specific cure has, in my opinion, caused many teachers and practitioners of the day to neglect the study of the effects of the various medicinal forms of mercury. When a case of syphilis presents itself, they apparently think that it is only necessary to saturate the system with the drug, regardless of the form of the remedy used or of the stage of the disease presented. The protoiodide is recommended by some authors in all stages of the disease and is given in increasing doses until a very large amount is taken during the day. In my own experience it has often been shown to my satisfaction that the pills and the tablets are sometimes so old and hard that they are not absorbed, but, acting as a local irritant, are expelled from the bowels, and that, when they are absorbed, the iodine eruption frequently complicates and increases that of the disease. In Alaska among the miners who came down from the gold mines were many cases of syphilis; some of these had an eruption resembling syphilitic rupia; all were much emaciated; two or three had symptoms of brain involvement; all had extreme gastro-intestinal irritation. These unfortunates had not had proper food, but peddlers had supplied them plentifully with nostrums warranted to cure syphilis, and they had taken them in large quantities. Analysis has shown that nostrums of this sort contain, as active ingredients, large quantities of potas. iodide and hydrag. chlord. corros., and I am persuaded that the men were suffering more from the effects of overdoses of these drugs than from syphilis. I treated an Indian in Sitka and gave advice to others, which apparently resulted in prompt relief, who had what appeared to be syphilitic rupia; he had been taking for months what was labeled "Hood's Sarsaparilla." To the taste this appeared to be a strong solution of potas. iodid. The symptoms promptly disappeared after the medicine was stopped and a simple tonic with ammon. mur. given.

The physiological action of potas. iodid. is well known and it is such that the action of the drug should be watched and it should be given in very small doses in the early stages of syphilis. In small doses the tonic effect, as well as the alterative, is well marked. It is also supposed to increase the effect and prevent the retention of mercury in the system. It is the contention of the writer that the iodide of mercury should not be given, or given very sparingly, in the early stages of syphilis; that it is better to use the mild chloride or the metallic form in the ointment, or oleate. Observation shows that about one-eighth of a grain of calomel will be absorbed in six hours, and that its absorption is rendered more certain by trituration with sugar of milk; when the amount taken is absorbed, there is little danger of irritation of the digestive tract. This quantity can be given thrice daily for three or four days before there are signs of mercurialization. When these appear, it should be stopped and small doses of potas. iodid., not over three grains, in a bitter tonic with ammonium muriate should be given until all signs of mercurial poisoning have disappeared; then two days of rest should be allowed; then the calomel repeated, followed by the iodide as before. After the primary ulcer and first eruption have disappeared the old mixed treatment—potas. iodid., not over five grains, and hydrag. chlor. corros., not over one-eighth of a grain, with ammon. chloride and a bitter tonic—should be given, but this should be stopped once a month and a short course of the mild chloride given as above. The history of many cases, some of whom have since come under my observation, has convinced me of the good results of this method of handling. There is no claim to originality in this. It has the weight of sanction of many syphilographers. The method is cleanly and attracts no attention to the patient. It has been my experience with two or three patients whom I remember, and there may have been others, that calomel can be taken for two or three months without intermission and with the most beneficial results. Again it has occasionally happened that it can not be taken at all in sufficient quantities to affect the disease, and then the soluble or insoluble salts may be used hypodermically. The objection to this method is the pain, the formation of a scar or an abscess, and the occurrence of embolism of the lung; but it is frequently a very convenient method and followed only by the best results. Inunction with mercurial ointment or the oleate is especially useful in obstinate skin and glandular involvement. Mercurial vapor baths also are often of the greatest value.

Whatever the method of medication used it is my firm opinion that the proper results of treatment can not be obtained unless the patients abstain from alcohol and tobacco; also from anything which their experience has shown them will cause indigestion. For this reason it has long been my custom to cut off not only the two articles mentioned above, but also coffee, tea, pastry and the other articles generally bought from the bum-boat. Though one can not always enforce these rules, a thorough explanation of the reasons will cause many patients to comply with them cheerfully. It is also necessary during the administration of mercury and potas. iodid, to cause thorough emptying of the intestinal canal once a week, at least, with, preferably, the vegetable cathartics.

There is another point on which opinions differ and that is when constitutional treatment should begin. The majority of practitioners wait for constitutional symptoms to appear, claiming that both patient and physician should be positive of the presence of syphilis before beginning treatment. While the writer appreciates this view, it seems to him to be founded on mistaken ideas and to be altogether objectionable. The saturation of the system with the syphilitic poison may be very rapid. We have in hospital a young man in whom the initial lesion appeared in December, 1908, about three weeks after exposure, and who was transferred to the hospital January 11, 1909, with paralysis of the left side of the face and a profuse eruption. He began to improve rapidly under the hypodermic use of hydrag. succinamid, but soon objected to the pain. The sailor has generally read more or less "literature," by which he has been firmly impressed with the idea that syphilis is a "blood disease," and that ulcers on the penis are generally syphilitic, therefore he must take some nostrum recommended. If the medical officer does not give any medicine, the patient will get the nostrum; frequently he takes both. Moreover, if the patient takes mercury as soon as the primary sore appears, the subsequent course of the disease will be lighter, but the specific eruption will appear if the drug be stopped for a few days, and will sometimes appear while the remedy is being exhibited. If the primary sore disappears very quickly under mercury, it is a fair presumption that the case is syphilitic. It has long been the writer's custom when a patient appears at sick call with abrasions or chancroids to give a mercurial purge—i. e., one-eighth grain of calomel every four hours until there is thorough purgation, and then to give a bitter tonic, and to repeat this calomel once a week until the ulcers heal. If they become indurated, the mercurial course indicated above is begun. It is my firm opinion that the early exhibition of mercury and the proper dietetic regime will make it unnecessary to send so many patients to hospital or to excuse them from duty even.

Of course, the finding of the treponema, or spirochaete, as it is generally called, may be considered diagnostic proof, but it is hard to find and may not be the proper form.

Colonel Lambkin, of the British medical service, speaks very highly of the abortive power of soamin and arsetacin, arsenic compounds, in syphilis. The writer has had no experience with them. —*Medical Director M. H. Simons, U. S. N., in the Military Surgeon.*

